# A25 DETECTION OF BACTERIAL ADP-HEPTOSE BY INTESTINAL STEM CELLS DRIVES EPITHELIAL REGENERATION FROM PANETH CELL DEDIFFERENTIATION

**DOI:** 10.1093/jcag/gwad061.025

**Published:** 2024-02-14

**Authors:** S Goyal, S Gray-Owen, S E Girardin

**Affiliations:** Laboratory Medicine and Pathobiology, University of Toronto, Toronto, ON, Canada; Laboratory Medicine and Pathobiology, University of Toronto, Toronto, ON, Canada; Laboratory Medicine and Pathobiology, University of Toronto, Toronto, ON, Canada

## Abstract

NOT PUBLISHED AT AUTHOR’S REQUEST

Please acknowledge all funding agencies listed below

Funding Agencies

CCC, CIHR

